# Upregulation of Centromere Proteins as Potential Biomarkers for Esophageal Squamous Cell Carcinoma Diagnosis and Prognosis

**DOI:** 10.1155/2022/3758731

**Published:** 2022-04-20

**Authors:** Xiao Wang, Minshan Lai, Yue Wang, Ruihuan Chai, Nan Li, Ling Ou, Kai Zheng, Jieling Li, Guifeng Xu, Shaoqi Wang, Yun Dong, Shaoxiang Wang

**Affiliations:** ^1^Shenzhen People's Hospital, Second Clinical Medical College of Jinan University, First Affiliated Hospital of Southern University of Science and Technology, Shenzhen, China; ^2^School of Pharmaceutical Sciences, Shenzhen University Health Science Center, Shenzhen, China; ^3^Polytechnic Institute, Zhejiang University, Hangzhou, China; ^4^Department of Oncology, Hubei Provincial Corps Hospital, Chinese People Armed Police Forces, Wuhan, China

## Abstract

Esophageal squamous cell carcinoma (ESCC) has a high incidence and low survival rate, necessitating the identification of novel specific biomarkers. Centromere-associated proteins (CENPs) have been reported to be biomarkers for many cancers, but their roles in ESCC have seldom been investigated. Here, the potential clinical roles of CENPs in ESCC patients were demonstrated by a systematic bioinformatics analysis. Most CENP-encoding genes were differentially expressed between tumor and normal tissues. CENPA, CENPE, CENPF, CENPI, CENPM, CENPN, CENPQ, and CENPR were upregulated universally in the three datasets. Survival analysis demonstrated that high expression of CENPE and CENPQ was positively correlated with the outcomes of ESCC patients. The CENPE-based forecast model was more accurate than the tumor-node-metastasis (TNM) staging-based model, which was classified as stage I/II vs. III/IV. More importantly, the forecast model based on the commonly upregulated CENPs exhibited a much higher area under the curve (AUC) value (0.855) than the currently known TTL, ZNF750, AC016205.1, and BOLA3 biomarkers. The nomogram model integrating the CENPs, TNM stage, and sex was highly accurate in the prognosis of ESCC patients (AUC = 0.906). Besides, gene set enrichment analysis (GSEA) demonstrated that CENPE expression is significantly correlated with cell cycle, G2/M checkpoint, mitotic spindle, p53, etc. Finally, in validation experiments, we also found that CENPE and CENPQ were significantly overexpressed in esophageal cancer cells. Taken together, these results clearly suggest that CENPs are clinically promising diagnostic and prognostic biomarkers for ESCC patients.

## 1. Introduction

Esophageal cancer ranks seventh globally in terms of incidence and sixth in overall mortality [1]. Esophageal cancer is classified into two main subtypes: esophageal squamous cell carcinoma (ESCC) and adenocarcinoma (EAC). In parts of Asia and sub-Saharan Africa, nearly 90% of esophageal cancer cases are ESCC [2], suggesting that ESCC is the dominant subtype. Although a variety of diagnostic methods and multiple therapies for ESCC patients have proven to be effective, the 5-year overall survival (OS) rate is still <20% [2]. Several studies have suggested that long noncoding RNA (lncRNA), microRNA- (miR-) 375, and miR-483-5p might serve as potential biomarkers for ESCC [3–5]. However, the outcomes of ESCC patients are still not ideal. The identification of novel diagnostic and prognostic biomarkers for ESCC is urgently needed to improve the outcomes of ESCC patients.

Centromere-associated proteins (CENPs) are a group of proteins involved in kinetochore formation. CENPs contain 18 inner kinetochore-located proteins (CENPA, B, C, H, I, K, L, M, N, O, P, Q, R, S, T, U, W, and X) and two fibrous corona-located proteins (CENPE and CENPF) [6]. Malfunctions of kinetochore can cause aneuploidy [7], a well-known hallmark of human cancers. This has prompted the reasonable hypothesis that CENPs play critical roles in tumorigenesis. Indeed, many researchers have confirmed that the dysregulation of CENPs is significantly associated with cancer prognosis and may serve as a biomarker for non-small-cell lung cancer and breast cancer [8, 9]. Only two specific CENPs, CENPE and CENPF, have been reported to be possible prognostic biomarkers for ESCC [10, 11]. The other CENPs need to be further investigated. The focus of this paper is to find a more accurate model for ESCC than previously reported, such as ZNF750, TTL, AC016205.1, and BOLA3 [12, 13].

The Cancer Gene Atlas (TCGA) and Gene Expression Omnibus (GEO) databases have provided a lot of information about gene expression profiles and clinical data related to cancer patients. Bioinformatics analysis provides a number of strategies for cancer prevention and treatment [14]. Herein, we conducted a comprehensive analysis of the expression of all genes encoding CENPs to assess their clinical significance in ESCC using TCGA and GEO data. Differentially expressed genes (DEGs) were identified by comparing the expression differences of the CENP-encoding genes between the tumor and normal samples. The effects of the expression of overlapping DEGs from the three datasets on the survival of ESCC patients were determined in a survival analysis. Based on the expression of overlapping DEGs, forecast models were established to forecast the survival of patients with ESCC. Furthermore, we explored the underlying mechanisms using gene set enrichment analysis (GSEA) and coexpression network analysis (WGCNA). Our results suggest that CENPs are promising diagnostic and prognostic biomarkers for ESCC.

## 2. Materials and Methods

### 2.1. Patient Profiles

Profiles of ESCC patients were downloaded from TCGA (https://portal.gdc.cancer.gov/) and the GSE38129 and GSE20347 datasets from GEO (https://www.ncbi.nlm.nih.gov/geo/). In TCGA, the clinical information data of ESCC and EAC were downloaded together. The samples with incomplete information were removed, leaving 95 ESCC patient cases. In addition, there were 30 cases in GSE38129 and 17 cases in the GSE20347 dataset. Relationships between the clinicopathological characteristics and the OS of ESCC patients were determined using univariate analysis using SPSS software (version 23.0).

### 2.2. Analysis of Expression Difference

The gene expression profiles of CENPs were extracted from the three datasets. In TCGA, some of the 95 clinical samples lacked gene expression, and 81 tumor samples and 11 normal samples were left after selection. In GSE38129, there were 30 normal and 30 tumor samples. In GSE38129, there were 17 normal and 17 tumor samples. Differences in CENP expression between normal and tumor samples were analyzed by calculating log_2_ fold change (logFC), false discovery rate (FDR), and *P* values using edgeR and limma packages in R 3.5.1 software for TCGA and GEO data, respectively [15, 16]. Genes with FDR < 0.05, and *P* < 0.05 were identified as DEGs. Heatmaps, Venn diagrams, and boxplots were plotted using R.

### 2.3. Survival Analysis

According to the gene expression level, DEGs in tumor samples were divided into low and high expression groups. Gene expression levels were ranked from high to low levels, with the top 50% as the high expression group and the bottom 50% as the low expression group. OS curves were plotted using R software based on the Kaplan–Meier method [17]. Statistical significance was set at *P* < 0.05.

### 2.4. Establishment of the Forecast Model

The risk scores of each patient were calculated as the sum of the expression levels of each gene multiplied by its corresponding coefficient using multivariate Cox regression analysis in R software [18]. Based on the risk scores and survival analysis data, time-dependent receiver operating characteristic (ROC) curves were plotted using the “survivalROC,” “timeROC,” and “bootstrap” package of R software. Package “survival” was used for multivariate risk regression analysis through Cox proportional hazards model. Sensitivity was the ordinate for true positive rate, and 1-specific was the abscissa for false-positive rate. A nomogram for individual forecast was generated based on the risk score of the multigene model and clinical risk factors using R software [19].

### 2.5. Analysis of the Mechanism

In GSEA, expression profiles of tumor samples were divided into CENPE-low and CENPE-high groups, as defined by the median expression value of CENPE. Hallmark gene sets, Kyoto Encyclopedia of Genes and Genomes (KEGG) gene sets, and oncogenic signature gene sets were used as references. Enriched gene sets were identified using GSEA-3.0.jar (http://software.broadinstitute.org/gsea/downloads.jsp). Gene sets with FDR < 0.25 and *P* < 0.05 were considered statistically significant [20]. WGCNA was conducted to identify genes coexpressed with CENPs [21]. The visualized network was plotted using Cytoscape 3.6.1. Correlation analysis was performed using Pearson's correlation analysis with R software.

### 2.6. Validation of Cell Lines

The cell lines, including KYSE 30, 410, 450, 510, 520, and HDF (human dermal fibroblasts), were purchased from the Shanghai Cell Bank of the Chinese Academy of Sciences (Shanghai, China). The cells were cultured in RPMI 1640 medium (Gibco Life Sciences, USA) supplemented with 10% fetal bovine serum (Gibco Life Sciences), 100 U/mL penicillin (Gibco; Thermo Fisher Scientific, Inc., USA), and 100 *μ*g/mL streptomycin (Gibco; Thermo Fisher Scientific, Inc.) and were incubated at 37°C in a humidified incubator containing 5% CO_2_. In quantitative real-time PCR analysis, total RNA was extracted using the RNeasy®Mini Kit (QIAGEN, USA). RT-qPCR was performed using SYBR Premix Ex Taq II (TaKaRa BIO, Japan) in a LightCycler® Real-Time PCR System (Roche, Switzerland). The thermocycling conditions included reverse transcription at 50°C for 10 min and initial denaturation at 95°C for 3 min, followed by 40 cycles of denaturation at 95°C for 15 s, annealing, and extension at 60°C for 30 s. The data were calculated using the 2−*ΔΔ*Ct method. The forward and reverse primers for CENPE were 5′-CAGCAGAGAAGAATCACTTG-3′ and 5′-GTACCATTGTAGCCTTGTATG-3′ and for CENPQ were 5′-CAATACCATCTCAACTTCCTG-3′ and 5′-TGTAGTAATGCCAGACCTTC-3′. Histograms were drawn using the GraphPad Prism 8 software. Statistical significance was set at *P* < 0.05. The gene expression profile of CENPE in esophageal cell lines was extracted from the GSE23964 dataset (two normal esophageal epithelial cell lines and 14 ESCC cell lines). Gene expression difference analysis was performed using the limma package in R software.

## 3. Results

### 3.1. Clinical Characteristics of ESCC Patients in TCGA

This study explored the effect of CENPs on ESCC (Figure S1). The relationship between the clinical characteristics and OS of ESCC patients in TCGA was clarified by performing univariate Cox regression analysis. Due to the lack of survival data, the GSE38129 and GSE20347 datasets were only used in the expression difference analysis. Male sex, advanced tumor-node-metastasis (TNM) stage, and N2 and N3 stages were significantly associated with poor survival of ESCC patients (*P* = 0.020, *P* = 0.015, and *P* = 0.012, respectively; Table 1). Therefore, N stage, sex, and TNM stage may be potential risk factors for OS in patients with ESCC.

### 3.2. Expression of CENPs in ESCC

**Figure 1:**
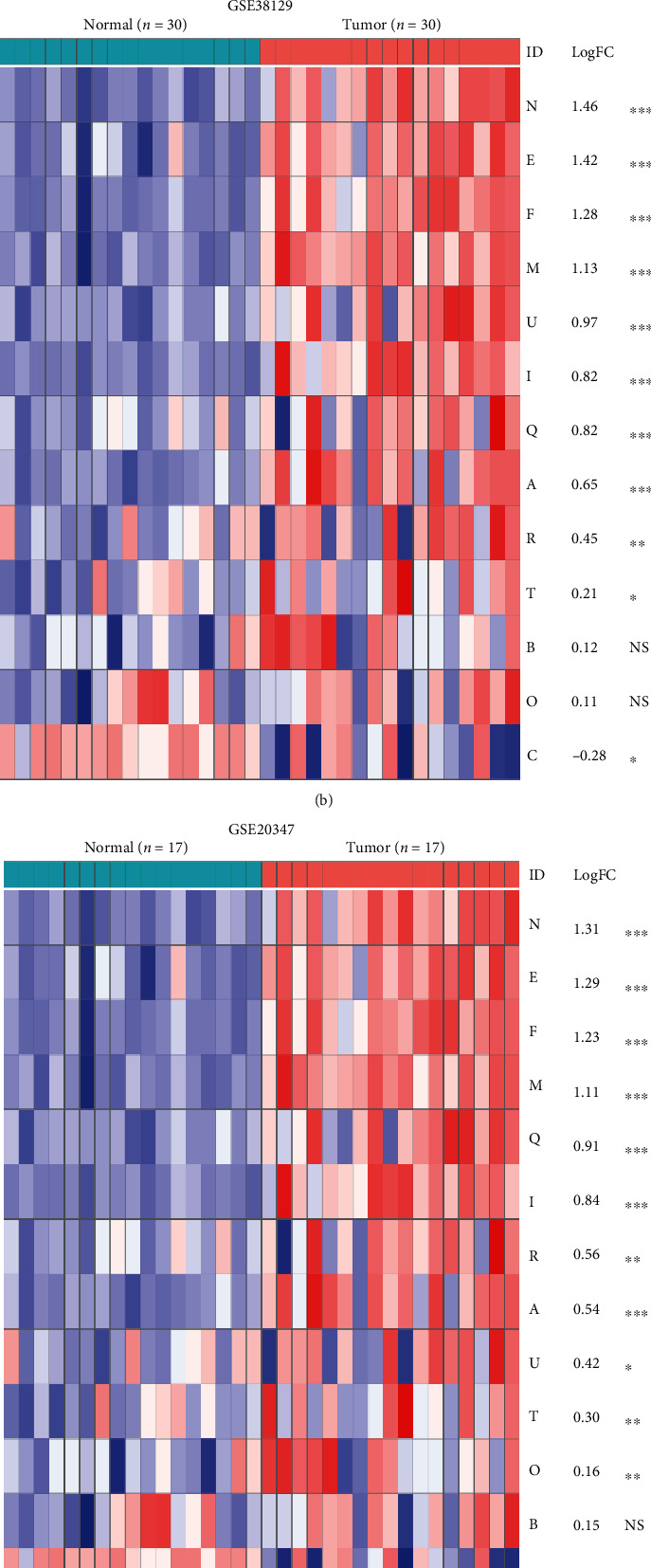
Aberrant expression of CENPs in ESCC. (a–c) Heatmaps showing expression differences of CENP-encoding genes between the tumor and normal samples in order of descending logFC in TCGA, GSE38129, and GSE20347. Blue and red colors represent low and high expression, respectively. ^∗∗∗^*P* < 0.001; ^∗∗^*P* < 0.01; ^∗^*P* < 0.05; and ^NS^*P* > 0.05. Genes with FDR < 0.05 and *P* < 0.05 were identified as DEGs. (d) Venn diagram displaying the overlapped DEGs in the three datasets, including CENPA, E, F, I, M, N, Q, and R. (e–g) Boxplots representing the different expression levels of the overlapped genes in tumor and normal samples according to TCGA, GSE38129, and GSE20347.

The expression levels of the CENP-encoding genes in tumor and normal samples were assessed to systematically identify the DEGs. The majority of CENPs were significantly aberrantly expressed in ESCC (17/20, 11/13, and 12/13 genes in TCGA, GSE38129, and GSE20347, respectively) (FDR < 0.05 and *P* < 0.05; Figures 1(a)–1(c)). While all the genes encoding CENPs seemed to be universally upregulated in ESCC (logFC > 0; Figures 1(a)–1(c)), the gene encoding CENPC was downregulated in GSE38129 and GSE20347 (logFC < 0; Figures 1(a)–1(c)). Among these DEGs, CENPA, CENPE, CENPF, CENPI, CENPM, CENPN, CENPQ, and CENPR overlapped in all three datasets (Figure 1(d)). The expression of the selected CENPs in each dataset is shown in boxplots (Figures 1(e)–1(g)), which clearly demonstrated a significantly higher expression profile in tumors than in normal tissues. The correlations among CENPs are shown in Figure S2. The collective findings revealed that a variety of CENPs might be promising diagnostic biomarkers for ESCC.

### 3.3. Correlation between Expression of CENPs and Survival of ESCC Patients

**Figure 2:**
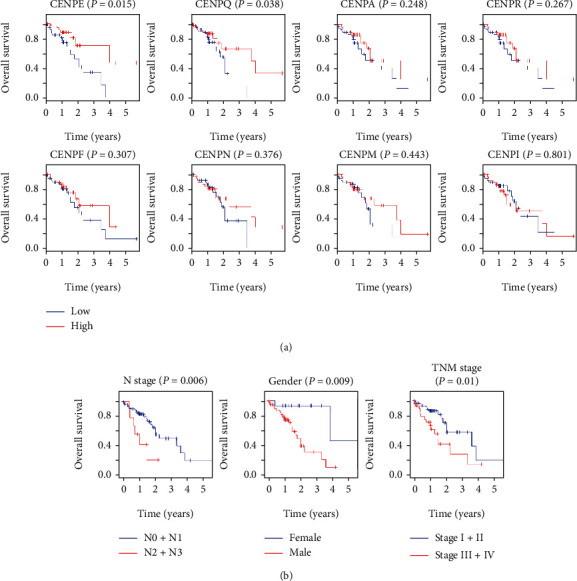
The Kaplan–Meier survival analysis of TCGA data. (a) OS curves of the overlapped DEGs, including CENPE, Q, A, R, F, N, M, and I in order of descending *P* values. (b) OS curves of three clinicopathological risk factors, N stage, sex, and TNM stage. The number of samples in (a) was 81. The total number of patients in (b) was 95. In N stage, N0 + N1 = 84, N2 + N3 = 9, and missing = 2. In gender, male = 80 and female = 15. In TNM stage, stage I + II = 63, stage III + IV = 31, and missing = 1.

The Kaplan–Meier OS curves were plotted to determine the prognostic value of the overlap of DEGs in ESCC patients. As shown in Figure 2(a), high expression levels of CENPE and CENPQ were significantly correlated with better outcomes in ESCC patients (*P* = 0.015 and *P* = 0.038, respectively). Although the other commonly upregulated CENP-encoding genes in three datasets (CENPA, CENPF, CENPI, CENPM, CENPN, and CENPR) did not display statistically significant survival differences between the lower- and higher-expressed groups (*P* > 0.05; Figure 2(a)), they exhibited a similar trend with CENPE and CENPQ, where high expression was associated with better survival. Therefore, CENPE and CENPQ may serve as potential prognostic biomarkers for patients with ESCC.

Since the N stage, sex, and TNM stage exhibited notable relationships with OS of ESCC patients in the univariate Cox regression analysis (Table 1), The Kaplan–Meier OS curves were also drawn for the three clinical characteristics. The results further demonstrated that N stage, sex, and TNM stage were tightly correlated with the OS of patients with ESCC (*P* = 0.006, 0.009, and 0.010, respectively; Figure 2(b)).

### 3.4. Prognostic Accuracies of CENPE, CENPQ, and the Other CENPs in ESCC Patients

**Figure 3:**
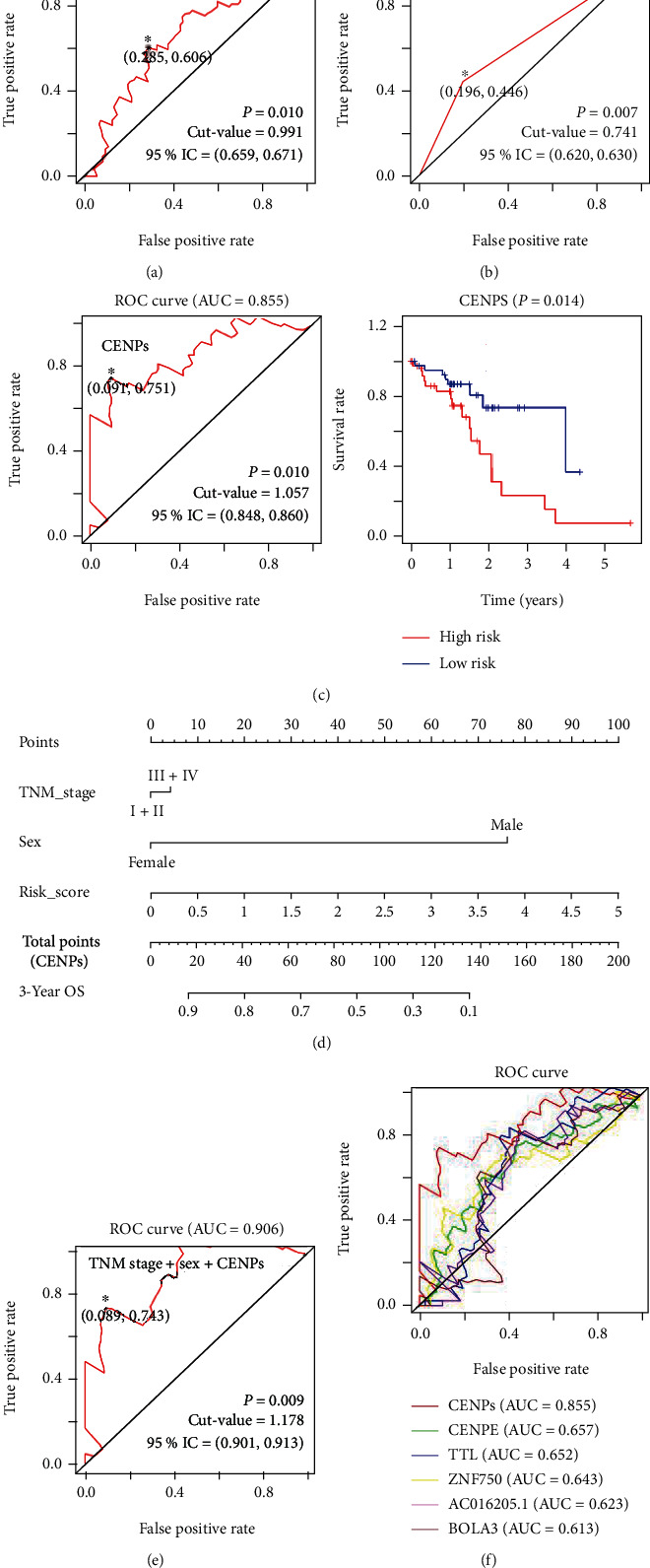
Forecast models to forecast the prognosis of ESCC patients. (a) Forecast model according to the TNM stage (I+II vs. III+IV) in ESCC. (b) CENPE-based forecast model. (c) Multigene forecast model and survival curve based on the expression of CENPs. (d) Nomogram integrating TNM stage, sex, and CENPs-based risk score to forecast individual OS for ESCC patients. (e) ROC curve to evaluate the nomogram's performance in forecasting patients' OS. (f) ROC curves showing the sensitivity and specificity of CENPE, CENPs, and other known biomarkers in forecasting ESCC patients' survival. The number of samples in (a) was 94, in (b, c) and (f) was 81, and in (d, e) was 79.

ROC analysis is a widely applied method to evaluate the prognostic performance of patients using the area under the curve (AUC) as an index [22]. The forecast model is significant only when its AUC value exceeds 0.60 [23–25]. By selecting those universally upregulated CENP-encoding genes, we established more focused forecast models to forecast OS. A single gene CENPE-based forecast model was more accurate than the TNM staging forecast model classified as stage I/II vs. III/IV in forecasting the OS of patients with ESCC (0.657 vs. 0.625, respectively; Figures 3(a) and 3(b)). However, the single gene forecast model of CENPQ did not show a superior value (AUC = 0.5). To assess the joint effect of these overlapping DEGs on patient survival, a multigene forecast model was established. Using the R package [18], the risk scores of patients were calculated according to the following formulas: Risk score = ( −0.020 × CENPA_Exp_ ) + (−0.966 × CENPE_Exp_) + (0.222 × CENPF_Exp_) + (0.899 × CENPI_Exp_) + (−0.520 × CENPM_Exp_) + (0.480 × CENPN_Exp_) + (−0.609 × CENPQ_Exp_) + (−0.402 × CENPR_Exp_).

The AUC value (0.8550 of such a multigene forecast model was satisfactory (Figure 3(c)) and was much higher than that of TNM staging, implying that the forecast model has high specificity and sensitivity for ESCC survival forecast. According to the median risk score, patients were divided into low-risk and high-risk groups. The corresponding survival curve demonstrated that low-risk patients had a higher survival rate than the high-risk group (*P* = 0.014; Figure 3(c)).

A nomogram is a reliable tool for the prognosis of cancer patients by incorporating and illustrating important factors for oncologic prognoses [26]. Based on the aforementioned results of the strong association of TNM stage and sex were tightly associated with patients with ESCC OS (Table 1 and Figure 2(b)), we further constructed a nomogram integrating CENP-based risk score and the two clinicopathological risk factors (TNM stage and sex). The N stage was excluded from the nomogram because it was included in the TNM stage. As shown in the nomogram, the CENP-based risk score contributed the most to forecast patients' OS, followed by sex and TNM stage (Figure 3(d)). The ROC curve showed that the true positive rate of our nomogram integrating CENP-based risk score, TNM stage, and sex could reach 90.6% (Figure 3(e)), implying the extremely high accuracy of the nomogram in forecasting individual OS of ESCC patients. In addition, to clarify whether our forecast models were superior in forecasting the survival of ESCC patients, compared with previously published biomarkers, we also performed ROC analysis of other known biomarkers, including ZNF750, TTL, AC016205.1, and BOLA3 [12, 13]. The results demonstrated that our CENPE-based forecast model, CENP-based forecast model, and integrated nomogram all had higher AUC values than the four other known biomarkers (AUC values for TTL, ZNF750, AC016205.1, and BOLA3 of 0.652, 0.643, 0.623, and 0.613, respectively; Figures 3(e) and 3(f)). Collectively, our forecast models based on CENPE, CENPs, and integrating CENP-based risk score, TNM stage, and sex are promising in the prognosis of ESCC patients.

### 3.5. Identification of the Potential Mechanism of CENPE in ESCC Progression

**Figure 4:**
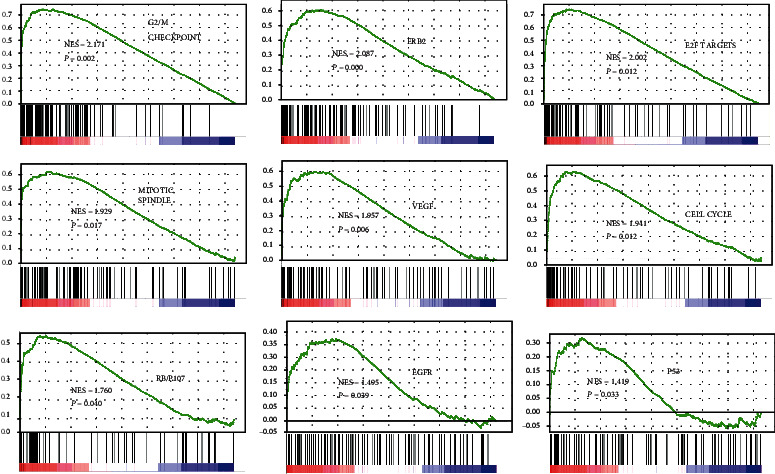
Significantly enriched gene sets in GSEA. The number of samples in GSEA was 81. NES: normalized enrichment score.

**Figure 5:**
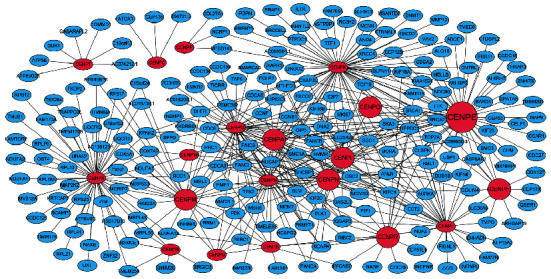
The coexpressed network of CENPs and their coexpressed genes. The blue circles represented the coexpressed genes. The red circles represented CENPs, of which the bigger ones represented the overlapped DEGs and the biggest one represented CENPE. The number of samples in WGCNA was 81.

To investigate the underlying mechanism of CENPE in ESCC progression, we performed GSEA and WGCNA. GSEA is a computational method to explore whether a specific gene set is markedly enriched in a group of gene markers ranked by their relationship to a phenotype of interest [20, 27]. In the experiment, the expression profiles of tumor samples were divided into CENPE-low and CENPE-high groups and then analyzed based on hallmark gene sets, KEGG gene sets, and oncogenic signature gene sets. Several cancer-related gene sets, including G2/M checkpoint, mitotic spindle, cell cycle, E2F targets, VEGF, RB/p107, EGFR, ERB2, and p53, were significantly enriched in the high CENPE expression group (FDR < 0.25 and *P* < 0.05; Figure 4). To further explore the molecular mechanism, we performed WGCNA and correlation analyses. In WGCNA, genes coexpressed with CENPs with correlation coefficients > 0.5 were selected and demonstrated in the visualized network (Figure 5). Some of the coexpressed genes that were highly related to CENPE and/or tumorigeneses, such as TOP2A, NDC80, BRCA1, CENPF, BARD1, TTK, BRCA2, and BUB1B, were further selected to plot correlation maps (Figure S3(a)). The results of differential expression analysis showed that BRCA1, BUB1B, and TTK were significantly upregulated in ESCC tissues based on the TCGA, GSE38129, and GSE20347 datasets, at the same time notable overexpressed in ESCC cell lines based on the GSE23964 dataset (FDR < 0.05 and *P* < 0.05; Figures S3(b)–S3(e)).

### 3.6. Target Validation in Human Cell Lines

**Figure 6:**
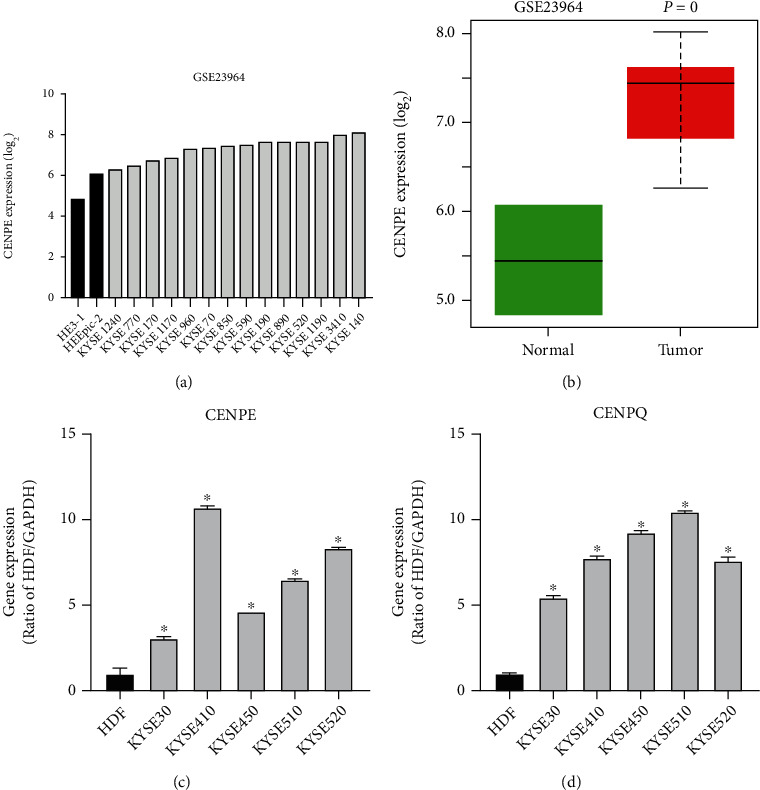
Validation of the mRNA expression differences of CENPE and CENPQ at the cell line level. (a) Histogram showing CENPE expression difference between ESCC cell lines and normal cells based on the GSE23964 dataset (2 normal esophageal normal epithelium normal cell lines and 14 ESCC ones). Normal cell lines and ESCC ones were filled in black and grey colors, respectively. (b) Boxplot representing CENPE expression difference based on the GSE23964 dataset. logFC = 1.86, *P* < 0.001. (c and d) RT-qPCR results of CENPE and CENPQ, respectively. Normal cell lines and ESCC cells were filled in black and grey colors, respectively. ^∗^*P* < 0.05.

To validate the mRNA expression differences of CENPE and CENPQ at the cell line level, RT-qPCR was done, and expression difference analysis was performed using expression profiles extracted from the GSE23964 dataset. Analysis results based on the GSE23964 dataset showed that CENPE was overexpressed by microarray assay in ESCC cell lines (logFC = 1.86, *P* < 0.001; Figures 6(a) and 6(b)). There were no CENPQ expression data in the GSE23964 dataset. In RT-qPCR analysis, since normal esophageal epithelial cell lines were very difficult to obtain, human dermal fibroblasts (HDF) were chosen as the ESCC control cell line with reference to other published articles [28, 29]. RT-qPCR analysis results demonstrated that both CENPE and CENPQ were significantly upregulated in the five ESCC cell lines compared to the normal cell line (*P* < 0.05; Figures 6(c) and 6(d)). Overall, these results validated that CENPE and CENPQ were upregulated in ESCC cell lines compared to the normal ones, consistent with their expression difference at the tissue level in TCGA and GEO databases.

## 4. Discussion

Tumor markers of esophageal cancer may have pivotal roles in evaluating tumor response to therapy [30], which could be exploited to develop early diagnostic biomarkers. Aberrant expression of CENPs has reportedly been related to several human cancers. For instance, in non-small-cell lung cancer, CENPU expression promotes cancer cell proliferation and forecasts poor survival [8]. In breast cancer, elevated expression of CENPA is associated with cancer malignant progression and is a prognostic biomarker [9]. However, the potential role of CENPs in ESCC has seldom been investigated. Herein, we systematically clarified the potential clinical functions of CENPs in ESCC patients using bioinformatics methods based on multiple datasets.

Most CENP-encoding genes, including CENPA, CENPE, CENPF, CENPI, CENPM, CENPN, CENPQ, and CENPR, were upregulated in ESCC patients in the TCGA, GSE38129, and GSE20347 datasets. Consistently, a previous study demonstrated the overexpression of CENPF in ESCC cell lines at both the mRNA and protein levels when compared to normal tissue [11]. CENPE was also upregulated in ESCC based on the TCGA dataset in a recent study [10]. However, the previous study only analyzed a single gene using a single dataset to draw the conclusion. In contrast, we obtained our results for all CENP-encoding genes based on three datasets, making it more convincing. Except for CENPE and CENPF, to the best of our knowledge, the overexpression of the other six CENPs in ESCC is described for the first time. Additionally, CENPH was overexpressed in ESCC samples compared to normal samples based on the TCGA dataset. Interestingly, CENPH is overexpressed and is prognostic in esophageal carcinoma [31].

In survival analysis, high expression of CENPE and CENPQ was significantly associated with better outcomes in ESCC patients. Similarly, some genes with high expression in pathological tissues will act against certain aberrations of pathological cells. Previous research has shown that the downregulation of CENPE causes an increase in aneuploidy, which in turn triggers an elevated level of spontaneous lymphomas and lung tumors in aged animals [32], implying that CENPE acts as a tumor suppressor. In a recent study, high expression of CENPE was closely correlated with better survival in ESCC patients but with unfavorable outcomes in EAC patients [10]. These findings indicate that CENPE might play crucial and complicated roles in the survival of cancer patients. Importantly, in the current study, CENPQ was first reported to act as a prognostic biomarker for ESCC patients. We speculate that although CENPE is highly expressed in tumor tissues, it has a good effect on survival, which may be a protective factor for ESCC. Thus, CENPE and CENPQ could serve as potential prognostic biomarkers for ESCC patients.

Since there are various factors that affect gene expression, a single gene is usually difficult to be an ideal factor to forecast. Indeed, the single gene CENPE-based model was capable of forecasting the OS of ESCC patients, while CENPQ was not. Therefore, a multigene forecast model based on the expression of CENPs was established. Satisfactorily, the multigene model exhibited an especially higher AUC value than that of TNM staging classified as stage I/II vs. III/IV, with an accuracy of 85.5% (AUC = 0.855). The stage I/II vs. III/IV based TNM staging system is a recognized benchmark for classifying the degree of spread of cancer and is a principal prognostic factor in forecasting the consequences of patients with cancer [33]. In this study, TNM staging was combined with the CENP-based risk score to construct a new nomogram-based forecast model. Surprisingly, the AUC value of the integrated nomogram reached 0.906, implying the high accuracy of our nomogram in the estimation of the individual OS of ESCC patients. Interestingly, the CENP-based risk score was the most important factor for OS in the nomogram to forecast, suggesting that CENPs are important in the prognosis of ESCC patients.

GSEA results demonstrated that cell cycle, G2/M checkpoint, mitotic spindle, RB/p107, p53, E2F targets, VEGF, ERB2, and EGFR were significantly related to CENPE expression. CENPE is a kinesin-like microtubule motor protein that accumulates maximally in the G2 phase [34]. It plays a crucial role in the cell cycle by forming a link between the attachment of spindle microtubules to kinetochores and the mitotic checkpoint [35]. High gene expression of CENPE is positively correlated with the tumor suppressor pathway. The G2/M checkpoint pathway can prevent the cell from entering mitosis (M phase). Hence, high CENPE expression inhibits cell division, which leads to a better prognosis in cancer patients. CENPE is regulated by E2F transcription factor 4. This regulation is important in maintaining G2-arrest and is regulated by p130/p107/Rb signaling [36]. Moreover, we found that CENPE was positively related to genes including TOP2A, NDC80, BRCA1, CENPF, BARD1, TTK, BRCA2, and BUB1B/BUBR1 by correlation analysis. CENPE, CENPF, TTK, and BUB1B are all mitotic spindle assembly checkpoint-related genes. The depletion of CENPE and CENPF has been related to the significant disruption of the cell cycle and paclitaxel resistance in ovarian cancer [37]. In addition, CENPE and TOP2A are upregulated in a number of solid cancers and are involved in mitotic cell cycle nodes in breast cancer [38]. Additionally, CENPE, TOP2A, CENPF, TTK, and NDC80 are highly expressed in the cell cycle of basal-like breast cancer [39]. Therefore, our findings indicate that CENPE affects ESCC progression, possibly by regulating cell cycle-related pathways.

In conclusion, CENPs, especially CENPA, CENPE, CENPF, CENPI, CENPM, CENPN, CENPQ, and CENPR, could serve as promising diagnostic biomarkers for ESCC. CENPE and CENPQ may be potential prognostic biomarkers for patients with ESCC. In addition, the CENPE-based model, CENP-based model, and nomogram integrating CENP-based risk score, TNM stage, and sex are especially promising in forecasting OS of ESCC patients. Mechanistically, CENPE may affect the progression of ESCC by regulating cell cycle-related pathways by interacting with TOP2A, NDC80, and BRCA1. Further studies are required to confirm their detailed roles by performing cell and animal experiments.

## Figures and Tables

**Table 1:** Univariate Cox regression analysis of ESCC patients' overall survival in TCGA.

Characteristics		Total *n* = 95*n* (%)	HR (95% CI)	*P*
Sex	Female vs. male	15 (15.8%) vs. 80 (84.2%)	0.175 (0.041-0.756)	0.020^∗^
Race	White+other vs. Asian Missing	47 (49.5%) vs. 45 (47.4%) 3 (3.2%)	1.570 (0.688-3.581)	0.284
Age	≥60 vs. <60	39 (41.1%) vs. 56 (58.9%)	1.296 (0.631-2.662)	0.461
T stage	T3+T4 vs. T1+T2 Missing	54 (56.8%) vs. 40 (42.1%) 1 (1.1%)	1.351 (0.649-2.811)	0.422
N stage	N2+N3 vs. N0+N1 Missing	9 (9.5%) vs. 84 (88.4%) 2 (2.1%)	3.265 (1.302-8.189)	0.012^∗^
TNM stage	III+IV vs. I+II Missing	31 (32.6%) vs. 63 (66.3%) 2 (2.1%)	2.443 (1.191-5.011)	0.015^∗^
Tumor grade	G3 vs. G1+G2 Missing	21 (22.1%) vs. 65 (68.4%) 9 (9.5%)	0.736 (0.277-1.950)	0.537
Tumor location	Lower vs. upper+middle Missing	44 (46.3%) vs. 50 (52.6%) 1 (1.1%)	0.958 (0.448-2.051)	0.913
Tobacco use	Yes vs. no	51 (53.7%) vs. 44 (46.3%)	1.965 (0.901-4.285)	0.089
Alcohol use	Yes vs. no Missing	68 (71.6%) vs. 25 (26.3%) 2 (2.1%)	2.172 (0.751-6.276)	0.152

HR: hazard ratio; CI: confidence interval; TNM: tumor-node-metastasis; ^∗^*P* < 0.05.

## Data Availability

The datasets analysed during the current study are available in The Cancer Gene Atlas (https://portal.gdc.cancer.gov/) and Gene Expression Omnibus (https://www.ncbi.nlm.nih.gov/geo/).

## Supplementary Materials

Supplementary Materials Figure S1: the flow chart of the study. From left to right, the leftmost row is all the original data, the contents in the dotted grid are the methods, then the right is the experimental results, and the rightmost row is the general name of the steps. Figure S2: Pearson's correlations among CENPs. Green color represented negative correlations and red color represented positive correlations. The deeper color indicated the higher correlations. Figure S3: (a) Pearson's correlations between CENPE and its coexpressed genes, including TOP2A, NDC80, BRCA1, CENPF, BARD1, TTK, BRCA2, and BUB1B. (b–d) Expression differences of the above 8 genes between the tumor and normal samples in TCGA, GSE38129, and GSE20347 datasets. ^∗∗∗^*P* < 0.001 and ^NS^*P* > 0.05. (e) Histograms showing expression differences of BRCA1, BUB1B, and TTK between ESCC cell lines and normal cells based on the GSE23964 dataset (2 normal esophageal normal epithelium normal cell lines and 14 ESCC ones). Normal cell lines and ESCC ones were filled in black and grey colors, respectively.
