# Supplementary material for: Upregulation of Centromere Proteins as Potential Biomarkers for Esophageal Squamous Cell Carcinoma Diagnosis and Prognosis
Source: Biomed Res Int. 2022 Apr 20;2022:3758731. doi: 10.1155/2022/3758731 (PMC9046002; doi:10.1155/2022/3758731)
Supplement: Supplementary Materials — Figure S1: the flow chart of the study. From left to right, the leftmost row is all the original data, the contents in the dotted grid are the methods, then the right is the experimental results, and the rightmost row is the general name of the steps. Figure S2: Pearson's correlations among CENPs. Green color represented negative correlations and red color represented positive correlations. The deeper color indicated the higher correlations. Figure S3: (a) Pearson's correlations between CENPE and its coexpressed genes, including TOP2A, NDC80, BRCA1, CENPF, BARD1, TTK, BRCA2, and BUB1B. (b–d) Expression differences of the above 8 genes between the tumor and normal samples in TCGA, GSE38129, and GSE20347 datasets. ∗∗∗P < 0.001 and NSP > 0.05. (e) Histograms showing expression differences of BRCA1, BUB1B, and TTK between ESCC cell lines and normal cells based on the GSE23964 dataset (2 normal esophageal normal epithelium normal cell lines and 14 ESCC ones). Normal cell lines and ESCC ones were filled in black and grey colors, respectively. [file 3758731.f1.zip › Supplementary Material (2).docx]

**Supplementary Material**

**Upregulation of centromere proteins as potential biomarkers for esophageal squamous cell carcinoma diagnosis and prognosis**

Shaoxiang Wang

Corresponding authors at:

S. Wang: wsx@szu.edu.cn; Tel./fax: +86-0755-86671959.

Figure S1: The flow chart of the study. From left to right, the leftmost row is all the original data, the contents in the dotted grid are the methods, then the right is the experimental results, and the rightmost row is the general name of the steps.

Figure S2: Pearson correlations among CENPs. Green color represented negative correlations and red color represented positive correlations. The deeper color indicated the higher correlations.

Figure S3: (a) Pearson correlations between CENPE and its co-expressed genes. The genes highly relating tumorigenesis and/or CNEPE were shown, including TOP2A, NDC80, BRCA1, CENPF, BARD1, TTK, BRCA2, and BUB1B. (b-d) Expression differences of the above 8 genes between the tumor and normal samples in TCGA, GSE38129 and GSE20347 datasets. ^***^P < 0.001; ^NS^P > 0.05. (e) Histograms showing expression differences of BRCA1, BUB1B, and TTK between ESCC cell lines and normal cells based on the GSE23964 dataset (2 normal esophageal normal epithelium normal cell lines and 14 ESCC ones). Normal cell lines and ESCC ones were filled in black and grey colors respectively.
